# Effect of Secondary Phase on Electroless Ni Plating Behaviour of Super Duplex Stainless Steel SAF2507 for Advanced Li-Ion Battery Case

**DOI:** 10.3390/ma17061441

**Published:** 2024-03-21

**Authors:** Byung-Hyun Shin, Seongjun Kim, Jinyong Park, Jung-Woo Ok, Doo-In Kim, Dohyung Kim, Jang-Hee Yoon

**Affiliations:** 1 Busan Centre, Korea Basic Science Institute, Busan 46742, Republic of Korea; lemonhouse211@kbsi.re.kr (B.-H.S.); seongjunk@kbsi.re.kr (S.K.); jinyongp@kbsi.re.kr (J.P.); jwok@kbsi.re.kr (J.-W.O.); 2 Innovative Graduate Education Program for Global High-Tech Materials and Parts, Pusan National University, Busan 46241, Republic of Korea; dooin.kim@pusan.ac.kr

**Keywords:** super duplex stainless steel, secondary phase, Li-ion battery case, electroless Ni plating

## Abstract

The development of Li-ion battery cases requires superior electrical conductivity, strength, and corrosion resistance for both cathode and anode to enhance safety and performance. Among the various battery case materials, super duplex stainless steel (SDSS), which is composed of austenite and ferrite as two-phase stainless steel, exhibits outstanding strength and corrosion resistance. However, stainless steel, which is an iron-based material, tends to have lower electrical conductivity. Nevertheless, nickel-plating SDSS can achieve excellent electrical conductivity, making it suitable for Li-ion battery cases. Therefore, this study analysed the plating behaviour of SDSS plates after nickel plating to leverage their exceptional strength and corrosion resistance. Electroless Ni plating was performed to analyse the plating behaviour, and the plating behaviour was studied with reference to different plating durations. Heat treatment was conducted at 1000 °C for one hour, followed by cooling at 50 °C/s. Post-heat treatment, the analysis of phases was executed using FE-SEM, EDS, and EPMA. Electroless Ni plating was performed at 60–300 s. The plating duration after the heat treatment was up to 300 s, and the behaviour of the materials was observed using FE-SEM. The phase analysis concerning different plating durations was conducted using XRD. Post-heat treatment, the precipitated secondary phases in SAF2507 were identified as Sigma, Chi, and CrN, approximating a 13% distribution. During the electroless Ni plating, the secondary phase exhibited a plating rate equivalent to that of ferrite, entirely plating at around 180 s. Further increments in plating time displayed growth of the plating layer from the austenite direction towards the ferrite, accompanied by a reduced influence from the substrate. Despite the differences in composition, both the secondary phase and austenite demonstrated comparable plating rates, showing that electroless Ni plating on SDSS was primarily influenced by the substrate, a finding which was primarily confirmed through phase analysis.

## 1. Introduction

The demand for Li-ion batteries is increasing owing to the increased demand for vehicles (electric cars) and electronic devices (phones, laptops, etc.) [1,2,3,4,5]. Various studies have been conducted aiming to enhance the performance of Li-ion batteries and ensure their safety [6,7,8]. Catalyst-related research has been widely pursued, seeking to improve Li-ion battery performance [9,10]. Additionally, the selection of the battery case is crucial, because it affects Li-ion battery performance. However, research into the development of case materials for secondary batteries is limited.

The structure of a Li-ion battery comprises a separator with a cathode and anode immersed in a Li-ion electrolyte solution [6,8,10]. The cathode requires excellent electrical conductivity and high corrosion resistance, whereas the anode necessitates high corrosion resistance [10,11,12,13]. Copper, which possesses excellent electrical conductivity, is used as the cathode in battery cases, whereas austenitic stainless steel (AISI304), with superior corrosion resistance, is employed as the anode [14,15,16]. Although copper exhibits superior electrical conductivity, it lacks strength [17,18]. Conversely, austenitic stainless steel provides excellent strength and corrosion resistance, yet exhibits lower electrical conductivity. Improving the conductivity of stainless steel is crucial for enhancing battery performance because low electrical conductivity hinders electron movement [13,19]. However, research on enhancing the electrical conductivity of stainless steel as an electrode material in Li-ion batteries is lacking and requires further investigation.

Stainless steel can be classified into various types based on its primary phase [20,21,22]. AISI304, predominantly composed of austenite owing to its high Ni content, belongs to the austenitic stainless steel category [21,23,24]. Austenitic stainless steel exhibits superior corrosion resistance yet lacks strength. Duplex stainless steel surpasses austenitic stainless steel in terms of both corrosion resistance and strength, making it beneficial for safety and enabling lightweight applications in Li-ion battery cases [25,26,27]. Duplex stainless steel is two-phase stainless steel consisting of both austenite and ferrite, categorised based on the pitting resistance equivalent (PRE = wt % Cr + 3.3 wt % Mo + 16 wt % N) [20,28,29,30]. A super-grade duplex stainless steel, such as SAF2507, with a PRE between 40 and 50, exhibits a strength of around 780 MPa and exceptional corrosion resistance. However, there have been no reported cases of SAF2507 being used in Li-ion battery cases.

SDSS SAF2507 is a material composed of austenite and ferrite dual-phase stainless steel, possessing high strength and a PRE of 42, owing to its high 25 wt % Cr and 7 wt % Ni content [31,32,33]. Because SAF2507 is duplex stainless steel, its chemical composition of austenite and ferrite varies depending on the heat treatment conditions (temperature and cooling rate), thereby optimising the corrosion resistance [34,35,36]. However, when the heat treatment temperature drops near 375 °C or 975 °C and is sustained for a certain time, SAF2507 precipitates a secondary phase [30,35,37]. This secondary phase, if allowed to form, can decrease corrosion resistance and must be controlled. Given that it can be generated by battery heat, an investigation into the application of SAF2507 in Li-ion batteries is required. However, the existing literature does not cover research on Ni plating in SAF2507’s secondary phase.

Ni plating can be wet or dry; wet plating, which is conducive to continuous production, is suitable for mass production [38,39,40]. Wet Ni plating is divided into electrolytic and electroless plating, based on the presence of electricity [41,42,43]. Electrolytic plating offers a rapid plating rate; however, it is highly influenced by the pre-treatment state. Electroless wet plating has a slower plating rate but allows for surface stabilisation before subsequent electroplating. Because research on electrolytic and electroless plating of SAF2507 is lacking, initial research on electroless plating before electrolytic plating is required.

Previous studies have extensively covered various aspects of SAF2507, including thermal treatments, addressed by J. Nilsson, aiming to determine the secondary phase precipitation temperature [20,44]. However, no research has focused on Ni plating. L. trinh and O. Beziou have studied SAF2507 welding [3,45]. With the recent surge in SDSS demand, numerous studies are underway. However, research specifically applying SAF2507 lacks a focus on Ni plating. D. Wang and H. Lee have investigated Ni plating on austenitic stainless steel [43,46,47]. However, further research is warranted because austenite and ferrite have different compositions and reactivities. Notably, there has been no prior research on Ni plating behaviour after precipitation of the secondary phase in SAF2507.

This study analysed the behaviour of electroless Ni plating over time using a Ni electrolyte solution, after precipitating the secondary phase through heat treatment of SAF2507 at 1000 °C. The distribution of the secondary phase was confirmed using FE-SEM and XRD. The composition of the precipitated secondary phase was analysed using EDS and EPMA. Electroless wet plating in a Ni electrolyte solution was conducted at intervals of 60–300 s. The behaviour of the Ni plating on SAF2507’s secondary phase was analysed using FE-SEM and XRD.

## 2. Materials and Methods

### 2.1. Materials

The material used in this study was SDSS SAF2507, a two-phase stainless steel composed of austenite and ferrite which is renowned for its excellent strength and corrosion resistance [20,44]. SDSS SAF2507 contains 25 wt % Cr and 7 wt % Ni, as indicated in Table 1. Additionally, SAF2507 includes alloying elements, such as Mo, Mn, and N, resulting in a high pitting-resistance equivalent number (PRE) of 42.

PRE = wt % Cr + 3.3 wt % Mo + 16 wt % N.(1)

A high alloy composition induces variations in the phase proportions based on the heat treatment conditions, consequently altering the chemical composition of the phases (austenite and ferrite). In iron, face-centred cubic (FCC) stabilising elements stabilise austenite, whereas base-centred cubic (BCC) stabilising elements stabilise ferrite.

### 2.2. Heat Treatment

**Figure 1:**
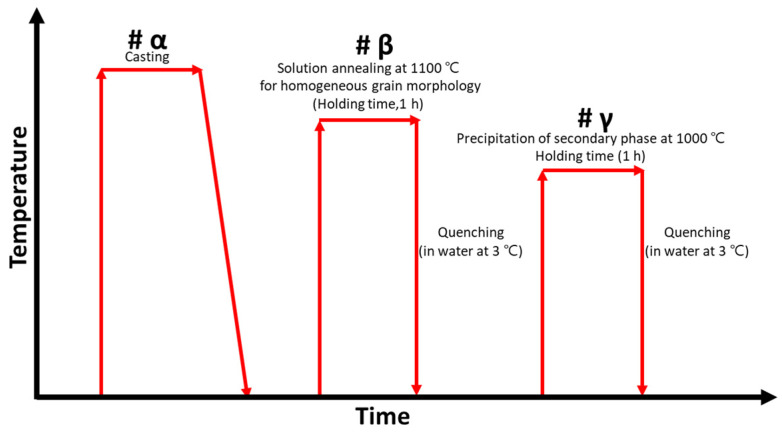
Schematic diagram of heat-treatment condition to precipitate secondary phase on super duplex stainless steel SAF2507.

To precipitate secondary phases in cast SDSS SAF2507, it was subjected to heat treatment at 1000 °C for 1 h, as depicted in Figure 1 [30,48]. The cast SDSS SAF2507, due to rapid cooling, exhibited a high austenite fraction of over 58%. Because uneven structures can induce high-temperature cracking, the cast SDSS SAF2507 underwent a solution annealing heat treatment for the precipitation of secondary phases.

Surface grinding and electrolytic etching were performed to examine the structure post-heat treatment. Surface grinding was conducted using abrasive papers from #100 to #2000, followed by 0.3 μm colloidal silica. Electrolytic etching was performed in a 10 wt % NaOH electrolyte solution at 10 V for 30 s [20,49,50]. Following etching, distinct phases were discerned, and the precipitated secondary phases were confirmed using FE-SEM (SUPRA 40VP system from Zeiss, Land Baden-Württemberg, Germany) and XRD (XRD, D8 VENTURE, Stanford, CA, USA). The phase proportions of SAF2507 post-treatment were analysed five times using image analysis (Image analyser 3.0) on ×200 magnified images [51]. The composition of the precipitated phases was determined by XRD, EDS (SUPRA 40VP system from Zeiss, Land Baden-Württemberg, Germany), and EPMA (Electron Probe Micro-Analysis, JEOL, Tokyo, Japan).

### 2.3. Electroless Ni Plating

**Figure 2:**
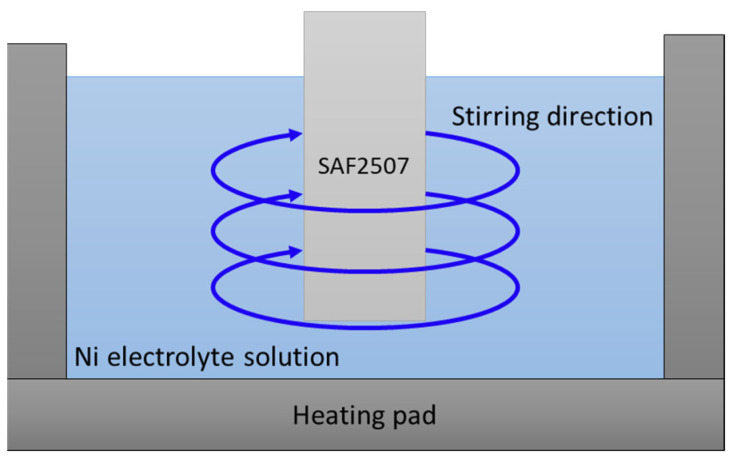
Schematic diagram of electrolyte bath for electroless Ni plating on super duplex stainless steel SAF2507.

Electroless Ni plating was conducted on heat-treated SDSS SAF2507, and the behaviour of electroless Ni plating over time in the Ni plating solution was analysed [43,46,47]. The surface post-heat treatment was polished using abrasive paper and colloidal silica. The plating process is illustrated in Figure 2. The plating solution comprised a mixture of hydrochloric acid (HCl, 37%) at 120 g/L and nickel chloride (NaCl_2_ + 6H_2_O) at 240 g/L. The behaviour of the electroless Ni plating over time was confirmed using FE-SEM and XRD. An electroless Ni plating solution was prepared by mixing 1 L of plating solution at 50 °C and 350 rpm. Electroless Ni plating was conducted in intervals of 60–300 s.

## 3. Results

### 3.1. Precipitation of Secondary Phase on SAF2507

**Figure 3:**
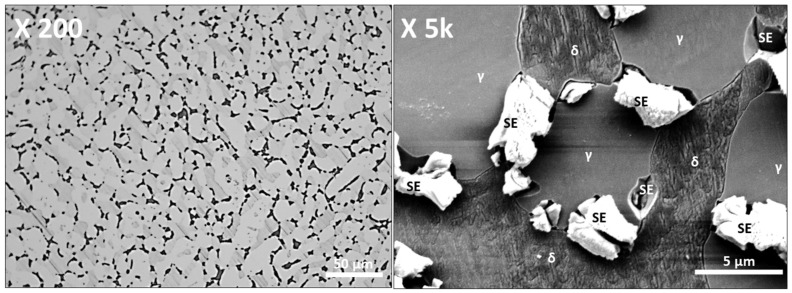
FE-SEM image after heat treatment at 1000 °C of super duplex stainless steel SAF2507.

SDSS SAF2507 demonstrated variations in the phase proportion based on heat treatment temperatures owing to its composition of austenite and ferrite, constituting a dual-phase stainless steel [20,48,52]. SAF2507 treated at 1000 °C precipitated secondary phases, as shown in Figure 3. At a low magnification of ×200, the secondary phases (SE) can be discerned as black dots, owing to differences in composition and brightness [30,37]. These appear to grow along the austenite and ferrite boundaries with indistinct morphologies. At a higher magnification of magnificent ×5000, the differentiation of the secondary phase, excluding austenite and ferrite, became clearer. Austenite appears bright grey, whereas ferrite appears darker. The secondary phase is divided into bright white areas, fine areas, and fine precipitates beneath, constituting three distinct zones.

Analysis after treatment at 1000 °C confirms the precipitation of secondary phases, with clearer differentiation at higher magnification. The secondary phase was separated into areas with higher resistance to wear and corrosion than shown by austenite and ferrite (unetched white portions), areas with lower corrosion resistance (corroded after etching), and remnants under etching. The secondary phase precipitated along the boundaries of austenite and ferrite and grew along the crystalline boundaries of the austenite. Phase ratios were determined as 56 ± 1.4% austenite, 30 ± 1.5% ferrite, and 13 ± 2.5% secondary phase, shifting from the initial 50:50 ratio of austenite to ferrite.

The proportion of the secondary phase was affected by the heat treatment temperature, as revealed by the EDS analysis detailed in Table 2. Elemental analysis of the secondary phase indicated a Cr-rich phase (Phase 1) containing over 30 wt % Cr and 9 wt % Mo, exhibiting a PRE close to 60 and superior corrosion resistance. The Cr-deficient phase (Phase 2) exhibited lower Cr and Mo contents, together with higher Ni and Mn contents, resulting in a PRE below 30, akin to the AISI316 levels of corrosion resistance. This lower resistance renders SAF2507 susceptible to passivation and galvanic corrosion, as is evident from electrolytic etching. Particles under 2 μm remain unetched and reside beneath phase 2, suggesting their precipitation is due to elements not integrated into Phases 1 and 2, and constituting corrosion-resistant precipitates.

**Figure 4:**
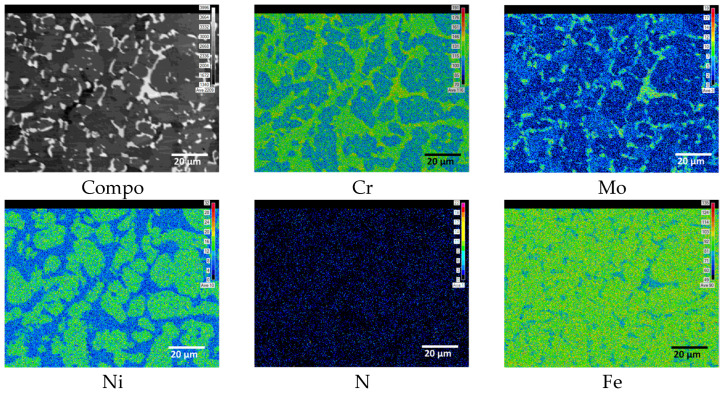
EPMA image of polished super duplex stainless steel SAF2507 from #100 to colloidal silica 0.25 μm after heat treatment at 1000 °C.

The distribution image concerning the composition was examined using EMPA to assess the impact of the secondary phase, as presented in Figure 4. The EPMA revealed higher concentrations of Cr and Mo in certain areas of the secondary phase. Ni and Fe appeared in lower concentrations within the secondary phase, whereas N seemed to be unincorporated. The EPMA analysis distinguishes the secondary phase as composed of two phases, in which the Cr-rich phase is apparent on the surface, whereas the Cr-deficient phase and particles from beneath the Cr-rich phase, remain absent from the EPMA results. The differing levels of Cr, Mo, and Ni contents in the secondary phase compared to those in ferrite and austenite allow for their separate identification.

**Figure 5:**
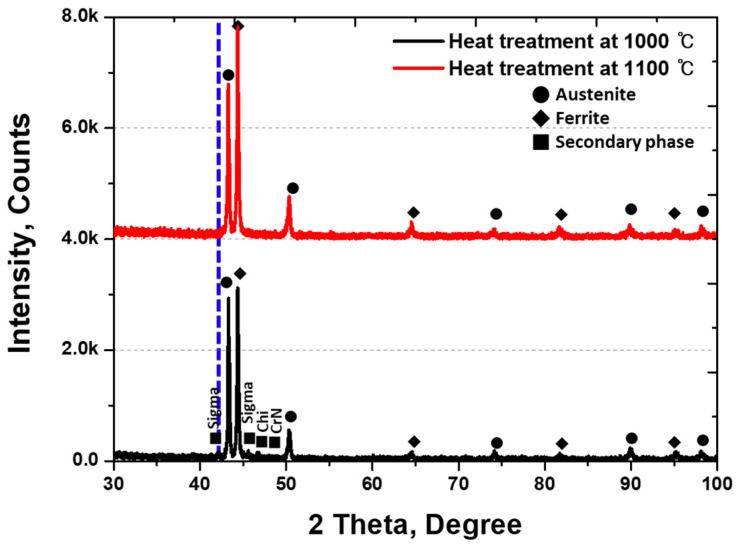
X-ray diffraction pattern with and without secondary phase after heat treatment temperature at 1000 °C and 1100 °C on super duplex stainless steel SAF2507.

An XRD analysis was conducted to examine the phase of austenite, ferrite, and secondary phase, as illustrated in Figure 5 [26,30,48]. The intensity of the secondary phase in XRD appears notably low owing to surface roughness, with the highest intensity being observed in austenite (at 56%), followed by ferrite (at 30%). Three patterns emerged in the secondary phase: Sigma, Chi, and CrN. Sigma represents Cr-rich Phase 1, and Chi signifies Cr-deficient Phase 2. CrN emerges as a precipitate at the boundary between Sigma and Chi owing to the unincorporated N. The secondary phase is divided into Sigma, Chi, and CrN, exhibiting distinct phases.

The secondary phase after heat treatment, with its differences in chemical composition and lattice structure from austenite and ferrite, results in varying reactivity during Ni plating, significantly influencing the plating capabilities.

### 3.2. Electroless Ni Plating Behaviour

**Figure 6:**
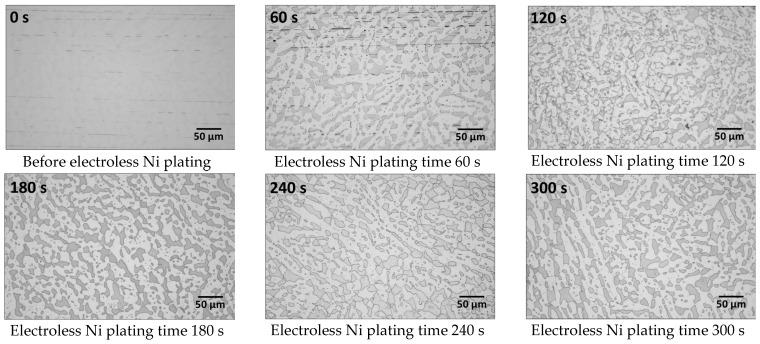
Surface image with electroless Ni plating, times from 0 s to 300 s, in Ni electrolyte solution of polished super duplex stainless steel SAF2507 after heat treatment at 1000 °C.

Investigation of the electroless Ni plating behaviour over time revealed an increase in the thickness of the plating layer. The phases were precipitated in SDSS SAF2507, and the electroless Ni plating behaviour over time was analysed. The plating behaviour with respect to electroless Ni plating time is illustrated in Figure 6. The SAF2507 surface underwent surface polishing using 0.3 μm colloidal silica before electroless Ni plating. The change in surface image according to electroless nickel plating time is shown in Figure 6. Two features were observed during the initial 0–180 s of the plating. Initially, the Ni plating layer differentiated between austenite and ferrite. However, the Ni-plating layer on the secondary phase did not show differentiation between austenite and ferrite. When the plating time was increased to 300 s, the portion appearing as ferrite decreased. Electroless Ni plating made it impossible to distinguish the secondary phase. This suggests equivalent Ni plating rates for austenite and ferrite.

**Figure 7:**
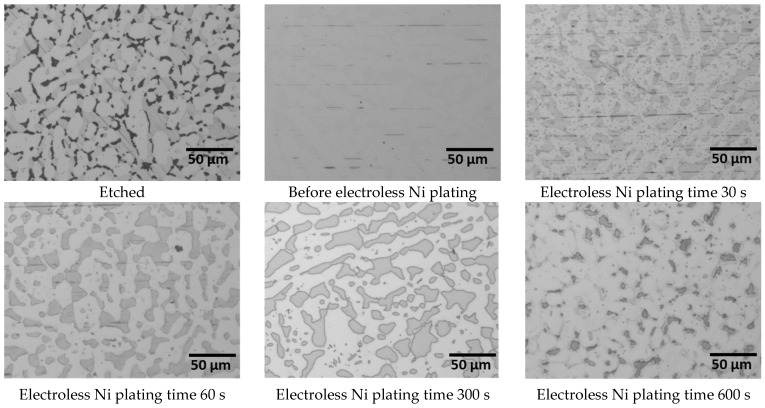
Surface image of etched and with electroless Ni plating, times from 0 s to 300 s, in Ni electrolyte solution of polished super duplex stainless steel SAF2507 after heat treatment at 1000 °C.

To examine the initial Ni plating behaviour, resolutions at ×5000 magnification were taken at 30 s intervals for up to 60 s, as illustrated in Figure 7. The initial Ni plating began with a reaction on austenite, followed by ferrite. While the surface polishing traces on the austenite mostly disappeared by 30 s, traces remained on the ferrite at 60 s. This indicated a preference for the initial reaction on austenite. At 30 s, fine grey images of 1 μm or less were observed within austenite during Ni plating. This suggests uneven growth during the initial stages of Ni plating, which evens out and disappears with increased plating time. Austenite demonstrated fast plating yet started growing unevenly before stabilising over time, whereas ferrite exhibited uniform but slower plating. Considering the phase proportions, the secondary phase initially showed a reactivity similar to that of austenite for up to 60 s but thereafter approached the reactivity of ferrite.

**Figure 8:**
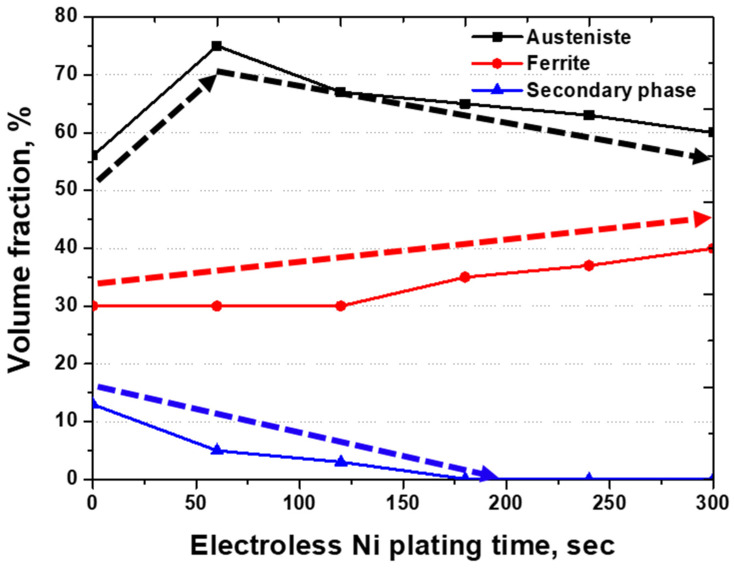
Volume fraction (%) vs. electroless curve, Ni plating, times (sec) from 0 s to 300 s, in Ni electrolyte solution of super duplex stainless steel SAF2507 after heat treatment at 1000 °C.

After analysing the surface images associated with the electroless Ni plating time, changes in the phase proportions were identified, as shown in Figure 8. The secondary phase and ferrite were indistinguishable, because both exhibited similar Ni plating rates, preventing differentiation as the plating time increased. Conversely, austenite, although not showing immediate changes in the early stages of plating, gradually increased in proportion over time.

The changes in the austenite and ferrite proportions can be attributed to the stabilisation of the plating layer with increasing plating time. The initial 60 s of Ni plating exhibited equivalent reactivity on austenite and the secondary phase. As the plating time increased beyond 120 s, the decrease in the proportion of austenite suggested the influence of the structure of the substrate. With further increases in plating time, the plating layer thickened, and the reduced influence of the substrate structure led to an increase in the austenite proportion. Up to the time-point of 600 s of electroless Ni plating, the Ni plating layer expanded from austenite to ferrite, indicating the decreased influence of the substrate structure and achievement of a more uniform plating layer.

**Figure 9:**
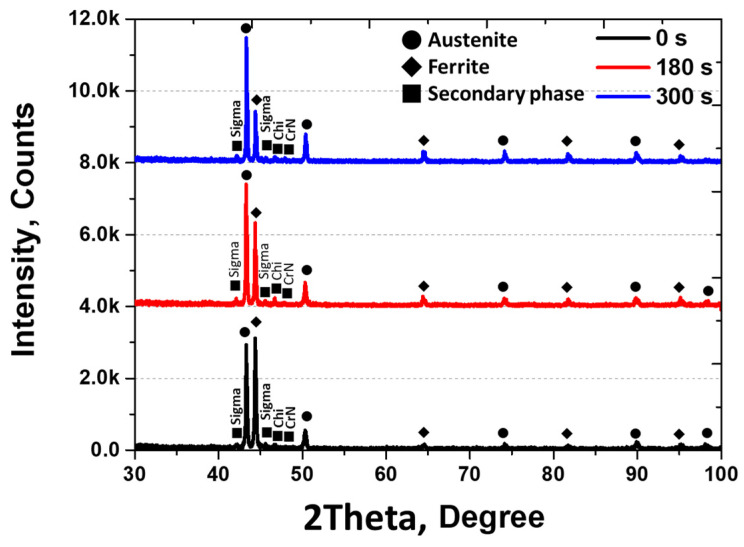
Intensity vs. degree curve, X-ray diffraction (XRD) pattern with electroless Ni plating time of super duplex stainless steel SAF2507 after heat treatment at 1000 °C.

X-ray diffraction (XRD) was employed to examine the plated state after electroless Ni plating on the secondary phase, and the results are shown in Figure 9. The Ni plating layer showed an increase in the intensity at the (111) plane, corresponding to austenite, at 42 degrees with increased plating time. Peaks corresponding to the secondary phase and ferrite were observed for up to 300 s of plating, followed by a decrease in the ferrite peak and an increase in the austenite peak with prolonged plating time. The increase in the austenite peak indicates an increase in the Ni plating layer. Although the peaks of the secondary phase and ferrite did not completely vanish due to the thickness of the Ni plating layer being below 10 µm, their influence increased.

**Figure 10:**
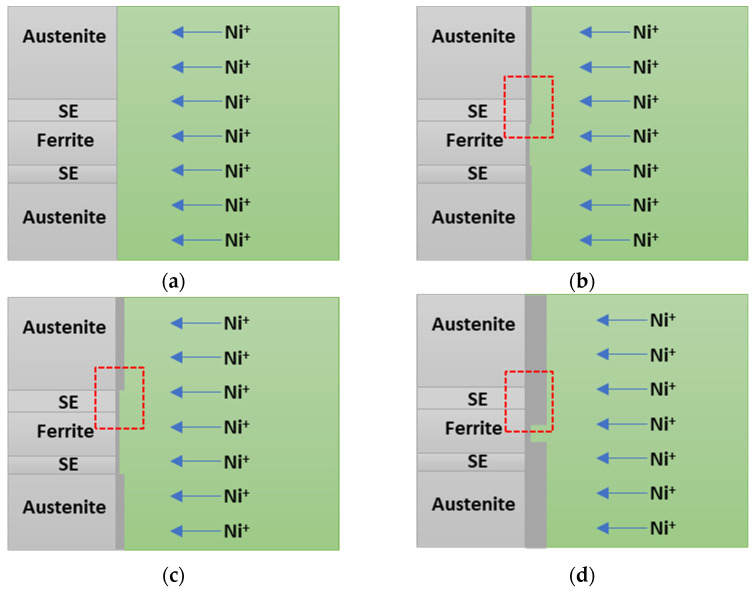
Electroless Ni plating behaviour with varying electroless Ni plating times of super duplex stainless steel SAF2507 after heat treatment at 1000 °C. (**a**) Before electroless Ni plating; (**b**) high range cover of austenite, ferrite, and secondary phase under 30 s of electroless Ni plating; (**c**) decreased cover range due to Ni by substrate effect under 300 s of electroless Ni plating; and (**d**) increased cover range of Ni due to decreased substrate and increased Ni layer thickness under 600 s of electroless Ni plating.

The electroless Ni plating mechanism over time is illustrated in Figure 10. Initially, Ni ions move from the solution towards the metal direction, initiating reactions [39,40]. As the plating progressed, Ni was deposited onto the substrate, but the rate varied depending on the phase [46,47]. Austenite, with an FCC structure similar to that of Ni, exhibited excellent plating speed, whereas ferrite and the secondary phase demonstrated slower plating. Contrary to the conventional literature suggesting that composition was a major factor influencing plating, in the case of SAF2507, the primary determinant appears to be the phase. With increasing electroless Ni plating time, a homogeneous Ni layer was formed, regardless of the phase, requiring a plating time beyond 600 s.

## 4. Discussion

To analyse the electroless Ni plating behaviour of super duplex stainless steel (SDSS) SAF2507 with precipitated secondary phases, heat treatment was conducted at 1000 °C [20,44]. Post-heat treatment, the proportion of the secondary phase increased to 13%, precipitating sigma, chi, and CrN. With a decrease in the temperature to 1000 °C, unutilised Cr in ferrite formed Sigma at the austenite boundary. Sigma, with 30 wt % Cr and 9 wt % Mo, depleted Cr, leading to the precipitation of Chi. Additionally, unutilised N in Sigma and Chi-formed CrN because it could not be accommodated in ferrite (with a nitrogen solubility limit of 0.05 wt %).

Electroless Ni plating on SDSS with precipitated secondary phases was controlled by the reactivity of Ni over time and the plating ability of the substrate [43,46,47]. Austenite demonstrated superior reactivity on the (111) plane. At 60 s, the austenite surface exhibited uniform plating, covering the surface imperfections. Although the secondary phase resembled austenite before 120 s, a subsequent increase in the proportion of ferrite was observed. Ferrite and the secondary phase exhibited reactivity with Ni, which influenced the plating layer. Variations in the electroless Ni plating rates between austenite and ferrite facilitated their differentiation. However, in reality, ferrite comprises the secondary phase, implying similar reactivity between ferrite and the secondary phase.

SDSS SAF2507, with precipitated secondary phases containing over 58% austenite, exhibited excellent electroless Ni plating capability. Higher proportions of austenite in SDSS led to reduced plating times. However, increasing the plating time affected the structure of the plating layer and decreased the proportion of austenite. Specifically, extending the plating time to 600 s resulted in the diffusion of the uniformly plated layer from austenite to ferrite and the secondary phase, thereby stabilising the Ni-plated layer.

When electroless Ni plating was conducted on SAF2507 with precipitated secondary phases, Ni in the electrolyte primarily reacted with austenite [38,39,40]. Ferrite exhibited the slowest reactivity, distinguishing itself from austenite. The secondary phase appeared to react primarily with the electrolyte containing Ni within the initial 60 s, showing a reactivity similar to that of austenite. However, after 60 s, it started showing an increased similarity to ferrite, increasing the proportion of ferrite. Nevertheless, with increasing plating time, both the secondary phase and the ferrite gradually underwent uniform plating. When the plating time reached 600 s, the influence of the substrate structure decreased notably, confirming the stabilisation of the Ni-plated layer.

Electroless Ni plating on SAF2507 with precipitated secondary phases exhibited excellent plating capability. Within the initial 30 s, the Ni plating layer covered both the austenite and the secondary phase, with subsequent influence from the structure of the substrate, leading to an increased ferrite proportion until 300 s. After approximately 600 s, the impact of the substrate diminished, forming a uniform Ni plating layer. The electroless Ni plating process is influenced by the structure of the substrate, with the secondary phase exhibiting effects similar to those of ferrite. As time progressed, the influence of the substrate structure decreased, with the secondary phase exerting a lesser impact than the ferrite.

## 5. Conclusions

These conclusions can be drawn from the analysis of the secondary phase precipitation and plating behaviour of super duplex stainless steel (SDSS) SAF2507 for advanced Li-ion battery cases:

(1) At 1000 °C, 13% of the secondary phase precipitated in SDSS SAF2507. This secondary phase precipitates at the austenite–ferrite interface and is driven by unutilised Cr and Mo during austenite growth. The secondary phase is composed of Sigma (Cr-rich phase, 30 wt.% Cr and 9 wt.% Mo), Chi (Cr-deficient phase, 22 wt.% Ni and 2 wt.% Mn), and CrN (Nitrogen not utilised in Sigma and Chi). The ratio of the secondary phase was 13% and showed variations in composition and phases.
(2) In electroless Ni plating, austenite exhibited excellent plating behaviour owing to the growth of the Ni plating layer on the (111) plane at 42°. During plating, ferrite and austenite were distinguished based on the differences in Ni plating rates. Initially (within 30 s), the secondary phase exhibits equal reactivity with austenite to 75%, followed by an increase in the proportion of ferrite to 40% at 300 s. Owing to their similar plating rates, the secondary phase and ferrite were indistinguishable. The differences in the electroless Ni plating rates were attributed to phase reactivity.
(3) SDSS SAF2507 is suitable for various applications, owing to its excellent strength and corrosion resistance. When used as a case for Li-ion batteries, it requires resistance against losses due to impact. SDSS possesses adequate strength, and when the secondary phase is precipitated, no decline in the plating ability is observed. Conversely, SAF2507 with precipitated secondary phase due to the presence of over 58% austenite exhibits superior plating ability, confirming excellent Ni plating ability for use in Li-ion battery cases. This study investigated electroless nickel plating on the secondary phase. However, further research is needed on the application of SAF2507 to Li-ion battery materials, including studies on VSM or electrochemical properties.

## Figures and Tables

**Table 1:** Chemical composition of casted super duplex stainless steel SAF2507.

Element	C	N	Mn	Ni	Cr	Mo	Cu	Fe
Chemical composition, wt %	0.01	0.27	0.8	6.8	25.2	3.8	0.2	Bal

**Table 2:** Chemical composition of polished secondary phase (Phases 1 and 2) from #100 to colloidal silica 0.25 μm after heat treatment at 1000 °C of super duplex stainless steel SAF2507.

Element	Cr	Mn	Ni	Mn	Fe
Phase 1	30.9 ± 1.5	8.9 ± 2.1	4.5 ± 0.8	0.5 ± 0.1	56.3
Phase 2	22.1 ± 0.9	2.2 ± 0.5	9.5 ± 1.2	1.0 ± 0.2	65.2

## Data Availability

Data are contained within the article.
